# LASSO Homotopy-Based Sparse Representation Classification for fNIRS-BCI

**DOI:** 10.3390/s22072575

**Published:** 2022-03-28

**Authors:** Asma Gulraiz, Noman Naseer, Hammad Nazeer, Muhammad Jawad Khan, Rayyan Azam Khan, Umar Shahbaz Khan

**Affiliations:** 1 Department of Mechatronics and Biomedical Engineering, Air University, Islamabad 44000, Pakistan; asmagulraizkiani@gmail.com (A.G.); hammad@mail.au.edu.pk (H.N.); 2 School of Mechanical and Manufacturing Engineering, National University of Science and Technology, Islamabad 44000, Pakistan; jawad.khan@smme.nust.edu.pk; 3 Department of Mechanical Engineering, University of Saskatchewan, Saskatoon, SK S7N 5A9, Canada; rayyan.khan@usask.ca; 4 Department of Mechatronics Engineering, National University of Sciences and Technology, H-12, Islamabad 44000, Pakistan; u.shahbaz@ceme.nust.edu.pk; 5 National Centre of Robotics and Automation (NCRA), Rawalpindi 46000, Pakistan

**Keywords:** BCI, fNIRS, SRC, channel selection, classification

## Abstract

Brain-computer interface (BCI) systems based on functional near-infrared spectroscopy (fNIRS) have been used as a way of facilitating communication between the brain and peripheral devices. The BCI provides an option to improve the walking pattern of people with poor walking dysfunction, by applying a rehabilitation process. A state-of-the-art step-wise BCI system includes data acquisition, pre-processing, channel selection, feature extraction, and classification. In fNIRS-based BCI (fNIRS-BCI), channel selection plays a vital role in enhancing the classification accuracy of the BCI problem. In this study, the concentration of blood oxygenation (*HbO*) in a resting state and in a walking state was used to decode the walking activity and the resting state of the subject, using channel selection by Least Absolute Shrinkage and Selection Operator (LASSO) homotopy-based sparse representation classification. The fNIRS signals of nine subjects were collected from the left hemisphere of the primary motor cortex. The subjects performed the task of walking on a treadmill for 10 s, followed by a 20 s rest. Appropriate filters were applied to the collected signals to remove motion artifacts and physiological noises. LASSO homotopy-based sparse representation was used to select the most significant channels, and then classification was performed to identify walking and resting states. For comparison, the statistical spatial features of mean, peak, variance, and skewness, and their combination, were used for classification. The classification results after channel selection were then compared with the classification based on the extracted features. The classifiers used for both methods were linear discrimination analysis (LDA), support vector machine (SVM), and logistic regression (LR). The study found that LASSO homotopy-based sparse representation classification successfully discriminated between the walking and resting states, with a better average classification accuracy (*p* < 0.016) of 91.32%. This research provides a step forward in improving the classification accuracy of fNIRS-BCI systems. The proposed methodology may also be used for rehabilitation purposes, such as controlling wheelchairs and prostheses, as well as an active rehabilitation training technique for patients with motor dysfunction.

## 1. Introduction

**Figure 1:**
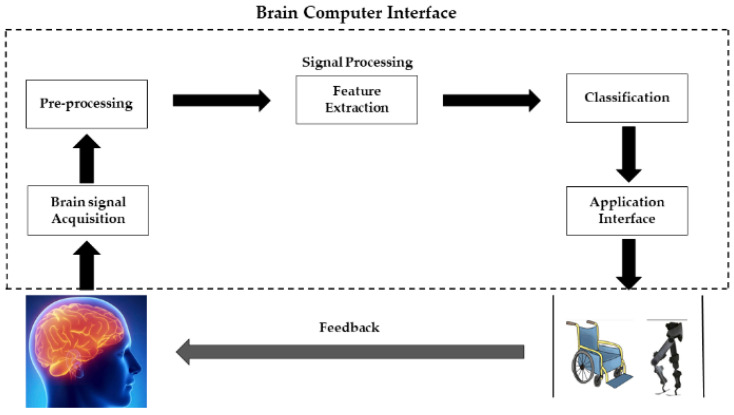
Block diagram of BCI system.

Nowadays, many elderly people have motor dysfunction and joint problems because of age factors, stroke, and spinal cord injuries. Due to this, they face many problems when walking, which strongly influences their lives [1]. According to WHO data, mental illnesses and neurological disorders are major sources of morbidity, death, and disability. Mental, neurological, and behavioral diseases have a major impact on the world’s population, impacting more than 450 million individuals. According to the Global Burden of Disease Report, neurological and mental disorders account for four out of the six primary causes of years lived with disability, accounting for 33 percent of the years lived with disability and 13 percent of disability-adjusted life years (DALYs) [2]. People with walking disabilities need to improve their walking patterns or capability by using rehabilitation and assistive devices [3]. The brain-computer interface (BCI) is the best way to accommodate the neuro-rehabilitation process, by providing a communication pathway between the brain and the peripheral devices [4]. The field of perceptible neuroscience scrutinizes itself with calibrating information processing models of the brain with the operational and structural (e.g., hemodynamic, metabolic, and electrical) features of the brain [5]. In the last few years, the development of BCI has played an important role in the analysis of brain dysfunction disorders and musculoskeletal gait. A typical BCI system consists of the following five major stages: signal acquisition, pre-processing, feature extraction, classification, and control commands to peripheral devices. In the signal acquisition stage, brain signals are acquired using brain signal acquisition modalities. The acquired signals contain noises such as physiological, instrumentation, and motion artifacts. These errors can be removed with the help of appropriate filters in the pre-processing stage. After obtaining filtered and processed data, some useful features can be extracted. These extracted features contain intrinsic information related to brain activity. In the classification stage, the most suitable classifier is employed for the extracted features, to predict the response to a particular class. Furthermore, the brain activity discriminated by the classifier is used as a command to control the external devices. The general flow diagram of functional near-infrared spectroscopy (fNIRS)-BCI is shown in Figure 1. BCI systems provide the end-user with full control over the channels used to communicate with the brain and external devices, regardless of the level of dependence on the output channel [1].

For improving mild cognitive impairment (MCI), BCI based on functional near-infrared spectroscopy (fNIRS) had a positive result [3]. It has been widely used in the rehabilitation process [6]. fNIRS is utilized to concentrate on the brain areas of interest in eleven sicknesses, including stroke, MCI, traumatic brain injury, and harm recognition [7]. There are several modalities used to acquire brain signals for rehabilitation, such as magnetic field measurement using magnetoencephalography (MEG) [8,9], electroencephalography (EEG), radioactive tracer-based positron emission tomography (PET) [10,11], functional magnetic resonance imaging (fMRI) [12,13], gamma emission-based single-photon emission computed tomography (SPECT) [14,15], and fNIRS. fNIRS is widely used due to its advantages of mobility and ease of use compared to other neuroimaging modalities when researching the brain basis of cognitive inputs during gait [16,17]. The fNIRS modality has been most commonly used over the recent decades, because of its portability and high spatial resolution. fNIRS is operated in wavelengths between 650 and 1000 nm; in this range, the blood oxygenation concentration (*HbO*) and the blood deoxygenation concentration (HbR) are more clear [7]. Several classifiers and techniques are applied to fNIRS signals [6,18], to improve the accuracy and efficiency of BCI systems, to help disabled and elderly people in their daily life [7,19].

For the classification of different brain activity, fNIRS-based BCI mostly extracted features such as mean, peak, variance, skewness, kurtosis values, etc., from the obtained data [20]. In the literature, studies have been performed using single, multiple or a combination of features to classify two- or multiple-class fNIRS-BCI problems [21]. Support vector machine (SVM) and linear discrimination analysis (LDA) are mainly used to classify walking and resting states, but the classification accuracy is low and needs to be improved [22].

**Figure 2:**
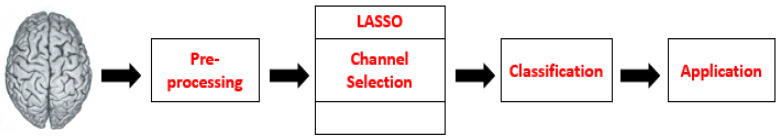
BCI system with LASSO-based sparse representation classification for channel selection.

To improve the classification accuracy, it is important to introduce some new methods and technologies in the field of fNIRS-BCI. In this study, a new classification method is discussed, which is sparse representation-based classification (SRC). SRC has been used in the compressed sensing (CS) theory; the core concept of CS is that we can represent a huge amount of data with a few data points [23]. Weighted SRC was applied to EEG-BCI to classify motor imagery, and achieved good classification accuracy results [24]. Sparse representation-based classification was used to translate the motor imagery of a single index finger classification, with an accuracy of 81.32%; the results were used to construct a BCI-enhanced finger rehabilitation system [25]. Optimization features, such as spatial-frequency-temporal, were calculated from the public dataset of EEG, and were used as predictors for SRC. The classification accuracy achieved was higher than on the original basis [26]. Shin et al. classified motor images using SRC and compared the results with SVM. They discovered that SRC had better results than SVM and LDA, in terms of classification accuracy, testing duration, and noise robustness [27]. This study includes the use of LASSO homotopy-based SRC for channel selection for the fNIRS-BCI system, to identify walking and resting states, Figure 2.

## 2. Materials and Methods

### 2.1. Experimental Design

The raw optical signals from the brain during activity and resting states were collected by dynamic near-infrared optical tomography (DYNOT; NIRx Medical Technologies, New York, NY, USA). For signal acquisition, the sampling frequency was set to 1.81 Hz, with operating wavelengths of 760 and 830 nm. A total of nine healthy male subjects, aged approximately 30 ± 3, were called up for the study. All the subjects were right-handed and had no neurological disorders. The experiments were conducted in accordance with the latest Declaration of Helsinki, and verbal consent from the subjects was collected before experimentation.

### 2.2. Experimental Paradigm

**Figure 3:**
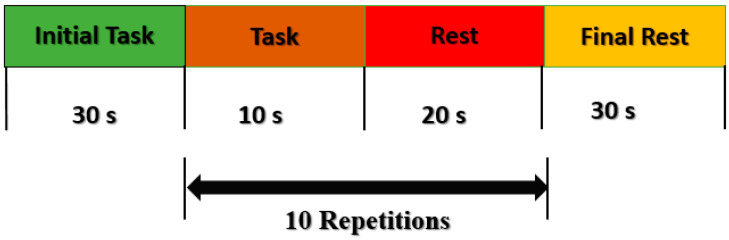
Experimental paradigm for data acquisition: after an initial 30 s rest, a single trial consisted of a 10 s period of walking followed by a 20 s rest.

The subjects were asked to take an initial rest in a quiet room for 30 s before the start of the activity. After the initial rest, subjects were asked to start walking with their right leg on the treadmill for 10 s, followed by a 20 s rest while standing on the treadmill. Ten trials were performed for each subject. For baseline correction, a 30 s rest was given at the end of each experiment. The length of the experiment for each subject was 300 s. The experimental paradigm is shown in Figure 3.

### 2.3. Experimental Configuration

**Figure 4:**
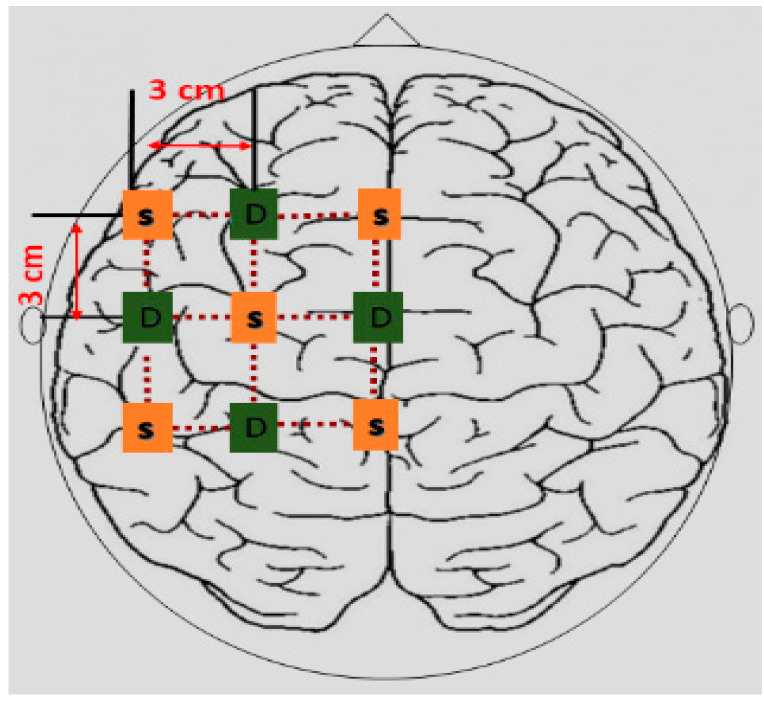
Position of source and detectors on the left hemisphere of the motor cortex. D represents the detectors and S represents sources.

In accordance with the literature [28], the twelve-channel configuration maintained a minimum distance distribution of 3 cm between the source and the detector. Brain signals from the left hemisphere of the primary motor cortex (M1) were acquired. There were nine optodes, out of which five were sources and four were light detectors. The configuration of the source and detector, with channels, is shown in Figure 4.

### 2.4. Data Acquisition

Raw optical density signals were converted into oxy and deoxyhemoglobin concentration changes ($\Delta C_{HbO}\left(t\right)$ and $\Delta C_{HbR}(t$) by using the modified Beer–Lambert law (MBLL) shown in Equation (1) [29].

(1) $$\left[\frac{\Delta C_{HbO}\left(t\right)}{\Delta C_{HbR}\left(t\right)}\right]=\frac{{\left[\begin{array}{cc} \alpha_{HbO}\left(\lambda_{1}\right) & \alpha_{HbR}\left(\lambda_{1}\right)\\ \alpha_{HbO}\left(\lambda_{2}\right) & \alpha_{HbR}\left(\lambda_{2}\right) \end{array}\right]}^{-1}\left[\begin{array}{l} \Delta A\left(t,\lambda_{1}\right)\\ \Delta A\left(t,\lambda_{2}\right) \end{array}\right]}{d\times l},$$

where $\alpha_{HbR}\left(\lambda_{1,2}\right)$ and $\alpha_{HbO}\left(\lambda_{1,2}\right)$ are the extinction coefficients of HbO and HbR in μM^−1^ cm^−1^, respectively, and $∆C_{HbR}\left(t\right)$ and $∆C_{HbO}\left(t\right)$ are the concentration changes in HbR and *HbO* in μM, respectively. Furthermore, l is the source and detector distance, *d* is the curved path length factor, and $A\left(t,\;\lambda_{1}\right)\;\mathrm{and}\;A\left(t,\;\lambda_{2}\right)$ are the absorption coefficients at two different instants.

### 2.5. Signal Processing

**Figure 5:**
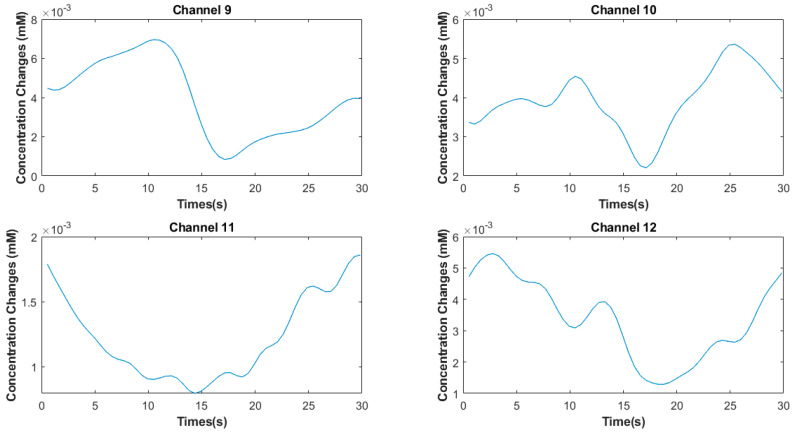
Average trial Δ*C_HbO_* signals of subject four for channels 9–12.

In this study, we only used the *HbO* response of brain activity for further processing. Noises including respiration between 1 and 1.5 Hz, heartbeat 0.5 Hz, and instrumental noise are present in the signals. These noises were removed using high-pass and low-pass filters with cut-off frequencies of 0.01 and 0.5 Hz [7]. The Hemodynamic Response filter and Gaussian filter were applied to the acquired signal for the removal of drift noise, using the NIRS-SPM toolbox [30]. For the motion artifacts, a hemodynamic response filter and discrete cosine transform were applied using the NIRS-SPM toolbox. Figure 5 shows the average trial $\Delta C_{HbR}\left(t\right)$ signals of subject four for channels 9–12.

### 2.6. Feature Extraction

A prior study explained several combinations of statistical features, with the goal of finding an effective filter for a given cortical region [31]. In this paper, spatial features were extracted from *HbO* data of all the active channels. The features were calculated for the entire task and rest session. The signal mean was calculated as follows:

(2) $$\mathrm{mean}=\frac{1}{N}\sum_{i=0}^{N}X_{i},$$

where the total number of observations is represented as *N*, and $X_{i}$ represents the $\Delta C_{HbO}\left(t\right)$ across each observation. The variance was calculated as follows:

(3) $$Var=\frac{\sum {\left(X_{i}-X\right)}^{2}}{n-1},$$

where *X_i_* represents the $∆C_{HbO}\left(t\right)$ across each observation, *X* is the mean value of observations, and *N* is the total number of observations. The *Skewness* was calculated as follows:

(4) $$Skewness=\frac{\sum {\left(X_{i}-\mu \right)}^{2}}{N\times \sigma },$$

where $X_{i}$ is each observation, μ is the mean of each observation, σ is the standard deviation of data, and N is the total number of observations. The peak values were calculated using the max function in MATLAB.

### 2.7. Channel Selection

Selecting channels of interest (COI) or a region of interest (ROI) in BCI can save processing time, reduce dimensionality, improve performance, and provide adequate brain region identification with low noise signals. In the literature, the *z*-score approach, which uses cross-correlation and *z*-scores for ROI/COI selection, was utilized to improve the performance of the fNIRS-BCI system [21]. The hemodynamic responses with positive *t*-values were selected by using the *t*-value method [32]. For pain-related cortical activations, the cross-correlation approach was employed to identify potentially dominating channels in both hemispheres. The response delay was calculated after a visual check, to identify probable dominating channels. The active channels that were next to each other were chosen [33]. In this paper, the LASSO homotopy-based sparse representation method is used for channel selection.

#### 2.7.1. Sparse Representation Classification

The basic idea of the SRC method is to recognize the true class of new signals by learning the sparsest representation (fewest significant coefficients) of the test signals, in terms of training signals [34]. A principle that a signal can be approximated by, using a linear combination of dictionary atoms, is formulated as follows [35]:

(5) $$\left(\frac{b}{A},\;x,\;k\right)=x_{1}a_{1}+\ldots +x_{k}a_{k}+\varepsilon ,$$

where the dictionary is represented as *A* = [*a*_1_, · · ·, *a_k_*], dictionary atom is represented as *a_i_*, *x* is a sparse coefficient vector, and ε is an error term. *A*, *x*, and k are the model parameters. In general, the SRC algorithm produces a dictionary before solving the optimization problem, reconstructing, and calculating the residual.

**Figure 6:**
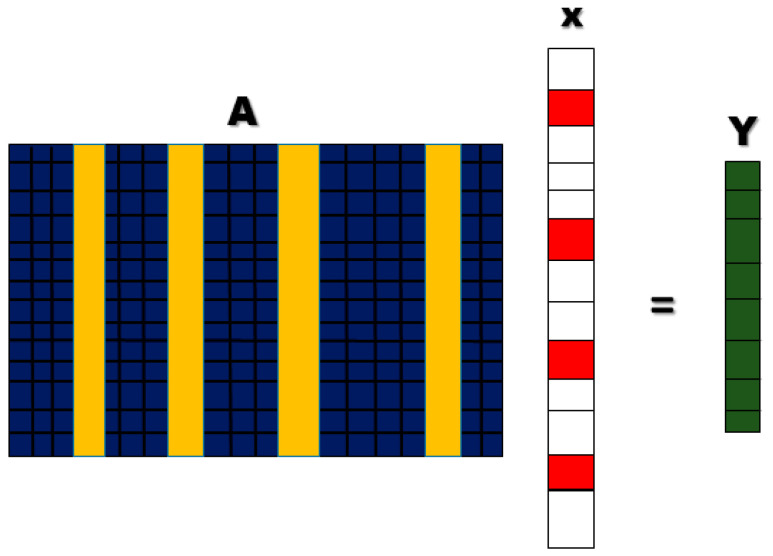
Sparse representation model. The dictionary is represented as *A* = [*a*_1_, ⋯, *a_k_*], dictionary atom is represented as *a_i_*, *x* is a sparse coefficient vector and Y is the output signal result as combination of *A ×* *x*.

For a certain category, when the residual is very small and the other categories are very large, the unknown category of the object belongs to that category [3]. The simplest sparse representation classification model is shown in Figure 6.

#### 2.7.2. LASSO Homotopy

The notion of homotopy comes from topology, and the homotopy technique is mostly used to solve problems involving nonlinear systems of equations. The homotopy approach was first developed to tackle the l_1_ penalty least squares problem [36]. Least absolute selection and shrinkage operator are representative approaches that use the homotopy-based strategy to tackle the sparse representation problem with l_1_-norm regularization (LASSO) [36]. Regularization is a crucial concept for avoiding data overfitting, especially when the learned and test data differ significantly. Regularization is implemented by adding a penalty term to the best fit produced from the trained data, in order to attain lower variance with the tested data, as well as by compressing the coefficients of the least important predictor or channel variable over the output variable. L_1_ regularization forces the weights of uninformative features and channels to be zero, by subtracting a small amount from the weight at each iteration, and, thus, making the weight of each channel or predictor equal to zero. LASSO homotopy starts optimization at a large value of *λ* parameter along the solution path and terminates at a point of *λ*, which is approximately zero, giving an optimal solution. The mathematical model of LASSO homotopy is represented as follows:

(6) $$\frac{1}{2N}{\left(y-X\beta^{\prime }\right)}^{\prime }\left(y-X\beta^{\prime }\right)+\lambda \;\sum_{j=1}^{P}\left|\beta_{j}\right|,$$

In the first term, *y* is the prediction value or test sample, X is the feature vector or trained sample, and $\beta^{\prime }$ is the vector of coefficients (weights on the basis of significance). The first term in the equation is the residual sum of squares (error term) and the second is product of *λ*× sum of the absolute values of the magnitude of coefficients (penalty term). *λ* denotes the amount of shrinkage. *λ* = 0 implies that all the features are considered and is equivalent to the linear regression, where the only residual square is considered to build a predictive model. *λ* = ∞ implies that no features are considered (i.e., as *λ* approaches infinity, it eliminates more and more features and channels).

### 2.8. Classification Algorithms

*K*-fold cross-validation is used to estimate classification performance. To ensure data separation for training and testing of classifiers for each channel selection method and activity utilized, the dataset was separated into training and testing sets, and the value of *k* was set to five-fold cross-validation.

In MATLAB^®^, the classification learner app was used for classification and validation of data. Several classifiers were selected and employed on the data, on the basis of prediction speed and training time. Following the literature [22], the following classifiers were used: linear discrimination analysis (LDA), logistic regression (LR), and support vector machine (SVM). The following settings were made during classification: covariance structure for LDA was set to diagonal covariance, and the kernel function for SVM was the Gaussian function.

## 3. Results

**Figure 7:**
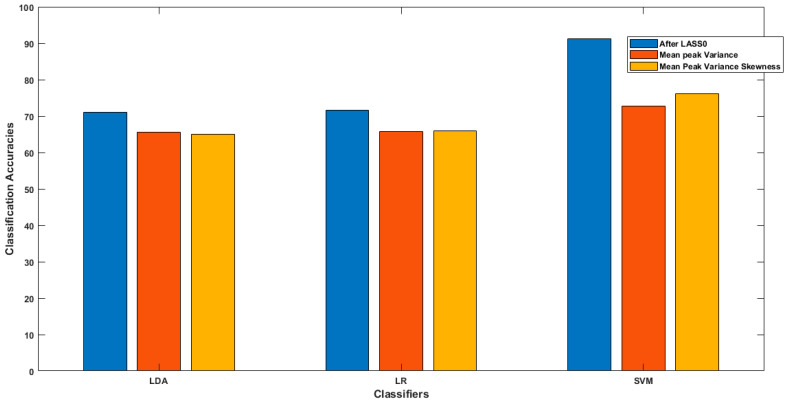
This figure shows a bar chart comparison of average classification accuracies for walking and resting states of all classifiers using both methods.

In this study, the LASSO homotopy method was employed for the channel selection of HbO signals with significant information; Table 1 shows the channels selected for each subject. From Table 1, we observe that the maximum and minimum channels selected by the LASSO homotopy method are nine and two for distinct subjects, respectively. The classification was performed using LDA, LR, and SVM on the data of the selected channels. The subject-wise average classification accuracies of all the classifiers used are given in Table 2. For comparison purpose, classification accuracies were calculated using conventional statistical features. Table 3 and Table 4 show the subject-wise classification accuracies of three- and four-feature combinations of statistical features. A comparison of the overall average classification accuracies of all the classifiers after channel selection using LASSO homotopy, and without channel selection, is shown in Table 5. In Table 6, the results of the *t*-test are shown [37]. A comparative bar graph is shown in Figure 7, for the average classification accuracies of all the classifiers.

## 4. Discussion

In the literature, recent studies have focused on enhancing the classification accuracies of fNIRS-BCI systems using the optimal classification technique [22], general linear model [38], vector-based phase analysis [38,39,40,41], optimal feature selection [31,38], optimal feature combination [42], *t*-value method [43,44], cross-correlation [45], and dominant channel selection [46]. An accurate and reliable fNIRS-BCI performance may lead to producing applications in neuro-robotics, rehabilitation, clinical BCI, for monitoring and analysis, and neuro-ergonomics.

In the present study, a new method for selecting channels on the basis of the strong influence of individual input variables on the output response was introduced to increase fNIRS-BCI performance, especially in terms of classification accuracy. In the literature, there were many optimization techniques used to enhance the classification accuracy of the fNIRS-BCI system, to make it more robust and reliable. A comparative analysis between classifications of fNIRS-BCI, based on two methods, was conducted. The classification accuracies based on the proposed method were compared with the accuracies based on the conventional method of excessively used feature extractions, without channel selection, using all the channel data. In the first method, we observed that by using two different combinations of spatial features, we achieved average classification accuracies, for LDA, LR, and SVM, of 65 ± 1.34%, 65 ± 1.6%, and 72 ± 4.9%, respectively. After the implementation of the other method, LASSO homotopy-based sparse representation for channel selection, the classification accuracies of LDA, LR, and SVM improved to 71.01, 71.6, and 91.32%, respectively. This study shows that selecting the channels with intrinsic brain information as features for classification improves the classification accuracy of fNIRS-BCI. LASSO homotopy-based SRC enhances both the prediction accuracy and model interpretability. It lowers the variability of the system estimations, by precisely decreasing some of the coefficients, and making models that are easy to understand, produce, and interpret [47]. For the channel selection method used for EEG-BCI, the classification accuracy was 93.08%, by selecting only eight channels out of 64 when classifying motor imagery tasks [48]. A similar study was performed to select cortical activation-based channel selection using the *z*-score method for fNIRS-BCI problems, achieving a classification accuracy of 88% [21]. LASSO homotopy-based SRC autonomously selects the most significant channels for the fNIRS-BCI system, thus greatly improving the overall classification accuracy.

This study has a few limitations, including the fact that it only applies to a single activity at a time, because specific tasks are linked to certain brain regions, and subject-based channels were selected due to the different brain sizes. LASSO homotopy-based SRC selects channels with the minimum residual sum of error. Furthermore, the offline study is performed and analyzed, while the online study may be conducted for other cognitive activities. Moreover, several machine learning algorithms are applied in this study to analyze performance. Further deep learning algorithms may be implemented with LASSO homotopy-based SRC for analysis, and may perform better.

## 5. Conclusions

This study attempts to apply LASSO homotopy-based sparse representation to fNIRS to identify the following two binary classes of data: walking state and resting state. The average classification accuracies are 71.01, 71.6, and 91.32% for LDA, LR, and SVM, respectively. The results show that LASSO homotopy-based SRC can effectively identify classes with significantly (*p* < 0.0167) improved classification accuracies. This study shows the better performance of LASSO homotopy-based SRC as a step to improve the classification performance of state-of-the-art fNIRS-BCI problems.

## Figures and Tables

**Table 1:** Subject-wise channel selection using LASSO homotopy-based spare representation.

Subjects	Selected Channels
1	1, 2, 3, 4, 7, 8, 9, 10, 11
2	2, 3, 4, 5, 6, 7, 9, 11
3	2, 6, 8, 9, 10, 11
4	8, 9, 12
5	1, 2, 5, 6, 7, 8, 12
6	1, 5, 8, 11, 12
7	2, 4, 5, 6, 8, 9, 11, 12
8	6, 10
9	1, 2, 3, 4, 6, 7, 8, 9

**Table 2:** Subject-wise classification accuracies of all subjects (%) were obtained by implementing LASSO homotopy for channel selection of HbO signals and classification using SVM, LDA, and LR of the walking and resting states (binary classification) of 9 subjects.

Subjects	LDA	LR	SVM
1	72.6%	69.1%	95.7%
2	75.7%	76.7%	95.9%
3	74.6%	83%	95.2%
4	68%	67.4%	85.4%
5	71.9%	72.4%	91.3%
6	68%	70.4%	95.2%
7	75.9%	74.6%	95.4%
8	62.6%	62.2%	75.9%
9	69.8%	69.8%	91.3%

**Table 3:** Subject-wise classification accuracies of all subjects (%) were obtained by extracting features (i.e., SM. SP, and SV) of HbO signals and classification using SVM, LDA, and LR of the walking and resting states (binary classification) of 9 subjects.

Subjects	LDA	LR	SVM
1	65.5%	63.9%	75.5%
2	66.5%	65.2%	72.4%
3	63.9%	62.8%	70.4%
4	66.9%	68.1%	68.9%
5	66.7%	66.7%	71.5%
6	61.9%	65.7%	71.3%
7	63.9%	64.8%	71.7%
8	66.5%	66.5%	71.7%
9	68.1%	67.4%	81.5%

**Table 4:** Subject-wise classification accuracies of all subjects (%) were obtained by extracting features (i.e., SM. SP, SV, and SK) of HbO signals and classification using SVM, LDA, and LR of the walking and resting states (binary classification) of 9 subjects.

Subjects	LDA	LR	SVM
1	65.4%	65.2%	78.1%
2	66.5%	69.4%	78.5%
3	64.6%	63%	71.9%
4	65%	65.9%	73%
5	66.1%	65.4%	74.8%
6	61.5%	65.9%	73.5%
7	62.8%	64.1%	72.6%
8	66.3%	68%	85.2%
9	67.6%	68%	85.2%

**Table 5:** Average classification accuracies of all subjects (%) were obtained by extracting features and selecting channels of HbO signals and classification using SVM, LDA, and LR of the walking and resting states (binary classification) of 9 subjects.

	LDA	LR	SVM
After LASSO Homotopy	71.01%	71.6%	91.32%
Mean, Peak and Variance	65.54%	65.67%	72.7%
Mean, Peak, Variance and Skewness	65.08%	65.9%	76.2%

**Table 6:** Statistical significance of the LASSO homotopy-based sparse representation method.

Bonferroni Correction Applied (*p* < 0.0167)			
**SVM**	**vs.**	**LDA**	1.0886 × 10^−6^
		LR	6.8421 × 10^−6^

## Data Availability

The original data used for this study can be shared upon reasonable request by the associate author.
